# A Multienzyme Reaction-Mediated Electrochemical Biosensor for Sensitive Detection of Organophosphorus Pesticides

**DOI:** 10.3390/bios14020062

**Published:** 2024-01-24

**Authors:** Chengzhen Ji, Xuemei Tang, Ruiming Wen, Chengdong Xu, Jing Wei, Bingjun Han, Long Wu

**Affiliations:** 1 Hainan Engineering Research Center of Aquatic Resources Efficient Utilization in South China Sea, Key Laboratory of Seafood Processing of Haikou, School of Food Science and Engineering, Hainan University, Haikou 570228, China; chengzhen.ji@outlook.com (C.J.); tangxm1996@163.com (X.T.); mchengdongxu@163.com (C.X.); 2 Key Laboratory of Tropical Fruits and Vegetables Quality and Safety for State Market Regulation, Hainan Institute for Food Control, Haikou 570314, China; weijing0128@126.com; 3 Hainan Provincial Key Laboratory of Quality and Safety for Tropical Fruits and Vegetables, Analysis and Test Center, Chinese Academy of Tropical Agricultural Sciences, Haikou 571101, China; hanbjun@163.com

**Keywords:** tropical fruits, organophosphorus pesticides, nanozymes, enzyme inhibition, food safety

## Abstract

Ethephon (ETH), a commonly employed growth regulator, poses potential health risks due to its residue in fruits and vegetables, leading to both acute and subchronic toxicity. However, the detection accuracy of ETH is compromised by the color effects of the samples during the detection process. In this work, a multienzyme reaction-mediated electrochemical biosensor (MRMEC) was developed for the sensitive, rapid, and color-interference-resistant determination of ETH. Nanozymes Fe_3_O_4_@Au–Pt and graphene nanocomplexes (GN–Au NPs) were prepared as catalysts and signal amplifiers for MRMEC. Acetylcholinesterase (AChE), acetylcholine (ACh), and choline oxidase (CHOx) form a cascade enzyme reaction to produce H_2_O_2_ in an electrolytic cell. Fe_3_O_4_@Au–Pt has excellent peroxidase-like activity and can catalyze the oxidation of 3,3′,5,5′-tetramethvlbenzidine (TMB) in the presence of H_2_O_2_, resulting in a decrease in the characteristic peak current of TMB. Based on the inhibitory effect of ETH on AChE, the differential pulse voltammetry (DPV) current signal of TMB was used to detect ETH, offering the limit of detection (LOD) of 2.01 nmol L^−1^. The MRMEC method effectively analyzed ETH levels in mangoes, showing satisfactory precision (coefficient of variations, 2.88–15.97%) and recovery rate (92.18–110.72%). This biosensor holds promise for detecting various organophosphorus pesticides in food samples.

## 1. Introduction

Ethephon (ETH) is a widely used growth regulator in fruits and vegetables [1]. It has a significant role in improving quality, promoting harvest [2], and improving the color of fruits and vegetables [3,4]. Thus, the consumption of fruits and vegetables increases the chances of humans ingesting excessive amounts of ETH. Several studies have shown that ETH has acute and subchronic toxic effects [5]. ETH can be introduced into the human body through dietary intake and skin absorption. It can result in corrosion of the gastrointestinal mucosa and digestive tract tissues, cause serious damage to the brain and kidney function [6], and produce a large anesthetic effect after entering the central nervous system. Real-time detection of ETH in fruits and vegetables can effectively reduce the risk of ETH poisoning. Traditional methods for the determination of ETH include gas chromatography-mass spectrometry (GC-MS) and high-performance liquid chromatography-mass spectrometry (HPLC-MS). For example, Niki et al. developed ion exchange chromatography for indirect detection of ETH in soluble concentrate formulations [7]. Kröpfl et al. developed a one-step derivatization procedure to improve the measurement accuracy of GC/MS analysis [8]. These methods, although highly sensitive, are subject to high background interference or have the disadvantages of high cost and large instrument size.

Since nanozymes were first discovered in 2004 [9], they have attracted a great deal of attention from scientists. According to the reaction mechanism, nanozymes can be divided into two main families: oxidoreductases and hydrolases, of which the oxidoreductase family of nanozymes includes peroxidases, oxidases, peroxidases, and superoxide dismutases [10]. Nanozymes are more stable, less susceptible to environmental influences, and easier to prepare than natural enzymes [11]. Moreover, nanozymes have stronger enzyme activity due to their larger specific surface area and more reactive sites. Nanozymes are often used in colorimetric assays [12,13,14]; however, the color of the sample itself may interfere with the assay results [15,16,17]. Therefore, it is of great significance to develop a new, rapid, sensitive, and convenient method for ETH detection. To solve the problem of color interference, electrochemical detection technology was introduced into the nanozyme sensor to replace the single colorimetric method. The electrochemical sensor not only has high sensitivity but also overcomes color interference. On the other side, electrochemical sensors are characterized by high speed, high sensitivity, simple operation, and low cost, but there is a low response signal at bare electrodes [18]. Graphene nanoparticles are two-dimensional honeycomb carbon materials with sp^2^ hybridized bonds, which have been frequently applied to improve electrochemical signals due to their merits of high specific surface area, electron mobility, thermal conductivity, and mechanical strength [19]. Notably, gold nanoparticles (Au NPs) modified graphene nanocomplexes exhibit more outstanding electrochemical sensing performance [20].

**Scheme 1:**
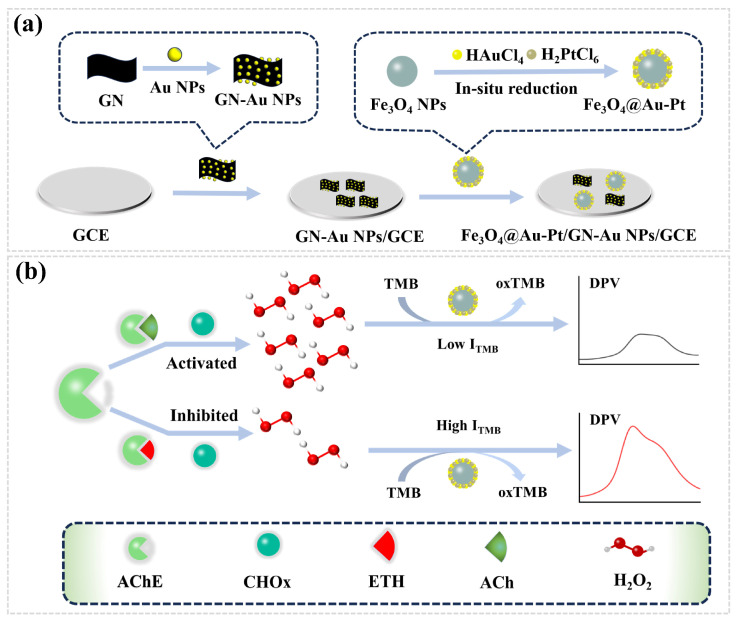
Schematic presentation of MRMEC for ETH: (**a**) modification process of GCE, (**b**) schematic diagram of enzyme inhibition and the differential pulse voltammetry (DPV) detection of ETH.

In this work, based on nanozymes catalysis and cascade enzyme reaction, an electrochemical biosensor was designed to detect ETH. As shown in Scheme 1a, GN–Au NPs composites were synthesized using a simple ultrasonic shock method and successfully modified on a glassy carbon electrode (GCE) to promote electric conductivity and generate enlarged response signals. Fe_3_O_4_@Au–Pt nanozymes with peroxidase-like activity were prepared using in situ reducing methods and immobilized onto the GCE to promote the catalysis of 3,3′,5,5′-tetramethvlbenzidine (TMB). As shown in Scheme 1b, the double-enzyme reaction is carried out in an electrolytic cell. Acetylcholinesterase (AChE) activity was inhibited in the presence of ETH, leading to less generation of H_2_O_2_. As a result, Fe_3_O_4_@Au–Pt cannot efficiently catalyze the conversion of TMB to oxTMB without enough H_2_O_2_, resulting in a higher current signal of TMB. Therefore, the concentration of ETH was positively related to the current signal of TMB. This electrochemical sensor not only avoids the color effect of the actual sample but also can be applied to other organophosphorus pesticides.

## 2. Materials and Methods

### 2.1. Chemicals and Instruments

Chloroauric acid trihydrate (HAuCl_4_, 4~50% Au basis), iron (II, III) oxide (MBs,100–200 nm, 5 mg mL^−1^), chloroplatinic acid (H_2_PtCl_6_, 99.9%), single-layer graphene oxide dispersion (2 mg mL^−1^, dispersion in H_2_O), acetylthiocholine iodide (ACH), acetylcholinesterase (AChE, 200 U g^−1^), and choline oxidase (CHOx, 8–20 U mg^−1^) were purchased from Shanghai Macklin Biochemical Co., Ltd. (Shanghai, China). Trisodium citrate dihydrate (C_6_H_5_Na_3_O_7_·2H_2_O), ethylenediaminetetraacetic acid disodium salt (C_10_H_14_N_2_Na_2_O_8_), citric acid monohydrate (C_6_H_10_O_8_), glycerol (C_3_H_8_O_3_), and dimethyl sulfoxide (DMSO, C_2_H_6_OS) were bought from Xilong Scientific Co., Ltd. (Guangzhou, China).

A 5500 UV-vis spectrometer (Shanghai Yuanxi Instrument Co., Ltd., Shanghai, China) was used to obtain UV-vis absorption spectra. All electrochemical measurements were performed on a CHI 440C (CH Instruments, Inc., Dellas, TX, USA) electrochemical workstation with a three-electrode system (a glassy carbon electrode, a platinum wire, and Ag/AgCl electrode were used as working, counter, and reference electrode, respectively).

### 2.2. Preparation of Fe_3_O_4_@Au–Pt

Fe_3_O_4_ was diluted with ultra-pure water in the ratio of 1:30. In the ultrasonic process, the diluted Fe_3_O_4_ solution was fixed at a constant volume of 5 mL and then introduced 200 μL HAuCl_4_ and 200 μL H_2_PtCl_6_, followed by adding 500 μL sodium borohydride (0.8 mol L^−1^) ice water solution. After the reaction, the aging process was carried out by standing still for 30 min.

### 2.3. Preparation of GN–Au NPs

A solution of sodium citrate (0.003 mmol L^−1^) was subjected to magnetic stirring for 5 min, and then 0.52 mL of 48 mmol L^−1^ HAuCl_4_ was added. Subsequently, 0.0114 g of NaBH_4_ was dissolved in ice water and slowly added to the above solution over a period of approximately 15 min until it turned wine-red. The resulting wine-red solution represents the initial gold nanoparticle solution. Then, 1 mL of single-layer graphene oxide dispersion solution and a small amount of NaBH_4_ (0.002 g) were added to an EP tube. After the reaction, 10 mL of Au NPs solution was added, and the mixture was sonicated for 30 min. 

### 2.4. Colorimetric Assay

The catalytic reaction was carried out in pH 7.4 buffer with 100 μL TMB and various concentrations of H_2_O_2_ as substrates. Then, 3 μL of the synthesized Fe_3_O_4_@Au–Pt was added, which can be used as an enzyme mimic. The mixed reaction solution was incubated at room temperature (25 °C) for 5 min. The total reaction volume was 1 mL.

### 2.5. Detection of ETH Using the Double-Enzyme Reaction System

First, the bare GCE was modified with 5 µL of GN–Au NPs and 5 µL of Fe_3_O_4_@Au–Pt, sequentially. Each modification was allowed to dry naturally for an equivalent duration each time. After the electrode modification was completed, the double-enzyme reaction was carried out in an electrolytic cell. A total of 50 µL ETH aqueous solution and 50 µL AChE (500 U L^−1^) were incubated at 37 °C for 30 min, then 50 µL ACh (5 mmol L^−1^) and 50 µL CHO (2000 U L^−1^) were successively added, followed by incubating the mixture for an extra 20 min. After the double-enzyme reaction, the reaction was started by adding TMB solution (1 mmol L^−1^) and phosphate buffer solution (PBS, pH = 7.4). Subsequently, the solution was exposed to electrochemical measurement, where the modified working electrode (Fe_3_O_4_@Au–Pt/GN–Au NPs/GCE) was used to detect TMB.

### 2.6. Detection of ETH in Real Samples

For real sample detection, mangoes from Hainan were selected for detection. Samples were sprayed with ETH aqueous solution (500, 50, and 5 μmol L^−1^) and ultrapure water. After waiting to dry naturally, a 10 g mango sample received the addition of 0.5 mL CH_3_OH-HCl and 50 mL CH_3_OH to obtain extraction solutions of spiked samples through ultrasound for 5 min. Mango residues were again extracted using 30 mL of CH_3_OH. The two extracts were combined to 100mL. The above operations were carried out in accordance with the requirements of Chinese national standards [21].

## 3. Results and Discussion

### 3.1. Characterization of Fe_3_O_4_@Au–Pt

**Figure 1:**
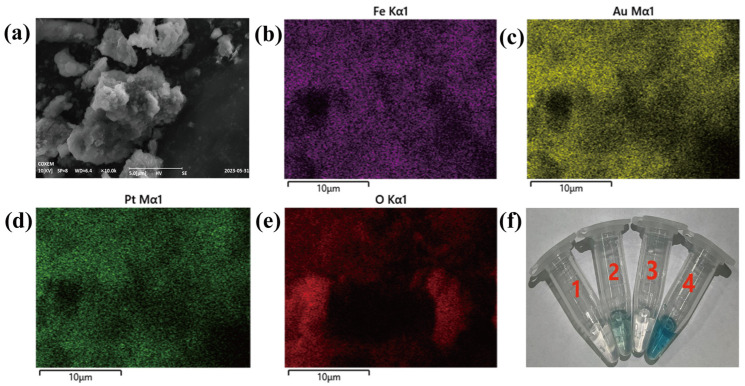
SEM images (**a**) and EDS elemental mapping pictures of Fe_3_O_4_@Au–Pt (**b**–**e**); chromogenic reaction diagram (**f**) (1: TMB, 2: TMB + H_2_O_2_, 3: TMB + H_2_O_2_, 4: TMB + H_2_O_2_ + Fe_3_O_4_@Au–Pt).

Schematic synthesis of Fe_3_O_4_@Au–Pt using the in situ reduction method [22] was shown in Figure S1, in which HAuCl_4_ and H_2_PtCl_6_ acted as an aurum and platinum source, and NaBH_4_ acted as a reducing agent. As illustrated in Figure 1a, Fe_3_O_4_@Au–Pt demonstrated an obvious spherical shape, enabling Fe_3_O_4_@Au–Pt to have a large specific surface area and larger active sites and promote higher enzyme-like catalytic activity. EDS elemental mapping pictures of Fe_3_O_4_@Au–Pt were shown in Figure 1b–e, which demonstrated that Fe_3_O_4_@Au–Pt simultaneously had Fe, O, Au, and Pt elements, and Fe elements were accompanied by Au and Pt elements. Furthermore, the content of O, Fe, Pt, and Au elements in the Fe_3_O_4_@Au–Pt reached 87.41%, 8.25%, 1.20%, and 3.14%, respectively (Figure S2). These results confirmed the preparation of Fe_3_O_4_@Au–Pt.

According to the peroxidase-like properties, our conclusions inferred that the prepared Fe_3_O_4_@Au–Pt could catalyze the oxidation of peroxidase substrates TMB in the presence of H_2_O_2_, causing a color change [23]. TMB lost electrons to transform into an oxidation product, and the colorless solution turned blue immediately [24]. The controlled experiment was carried out to prove the peroxidase-like property of Fe_3_O_4_@Au–Pt. As shown in Figure 1f, the effect of the Fe_3_O_4_@Au–Pt was first demonstrated using a color development experiment. Fe_3_O_4_@Au–Pt can still oxidize TMB in the absence of H_2_O_2_, turning the solution blue. Furthermore, the catalytic effect of Fe_3_O_4_@Au–Pt was presented by the UV-vis absorption spectrum (Figure S3a). The absorbance values were further enhanced after the addition of Fe_3_O_4_@Au–Pt to the TMB and mixed solutions of TMB and H_2_O_2_. Moreover, the catalytic activity of Fe_3_O_4_@Au–Pt was further investigated using electrochemistry. The electrochemical signals of Fe_3_O_4_@Au–Pt/GCE were consistently lower than those of GCE, both in the PBS (Ph = 7.4) (Figure S3b) and H_2_O_2_ solution (Figure S3c), and it is noteworthy that the electrochemical signals of Fe_3_O_4_@Au–Pt/GCE can decrease in a short period of time (Figure S3d). The above results confirm that Fe_3_O_4_@Au–Pt has better catalytic properties.

Based on the catalytic effect of Fe_3_O_4_@Au–Pt, increasing the concentration of H_2_O_2_, the peak intensity of UV absorption increased at 652 nm (Figure S4a). Figure S4b shown a good linear relationship between the absorbance and logarithm concentration within 0.05–6 mmol L^−1^. The working equation is Y = 0.0513 + 0.278X (R^2^ = 0.9803, n = 3), where Y and X were the absorbance and the logarithmic concentration of H_2_O_2_, respectively. 

The above-mentioned could lead to the conclusion that nanozyme was used in colorimetric methods and had a good linear relationship. However, for the detection of H_2_O_2_ in real samples, the colorimetric method suffered from low sensitivity and color interference.

### 3.2. Characterization of GN–Au NPs

**Figure 2:**
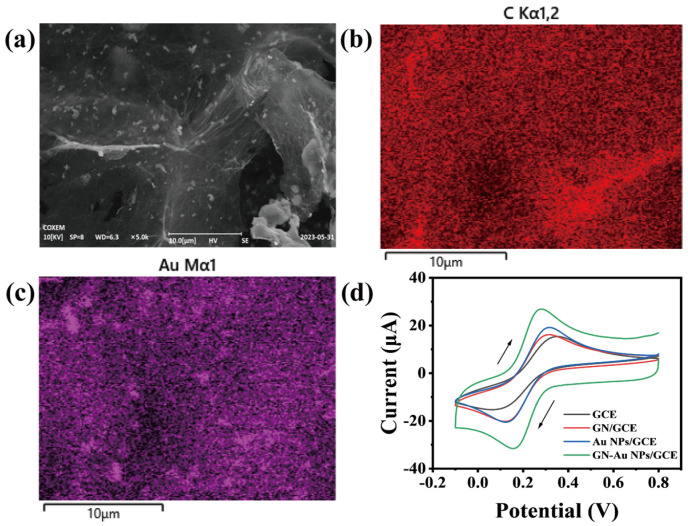
SEM images (**a**) and EDS elemental mapping pictures of GN–Au NPs (**b**,**c**). Cyclic voltammetry curve of bare GCE, GN/GCE, Au NPs/GCE, GN–Au NPs/GCE in 0.1 mol L^−1^ KCl solution containing 1.0 mmol L^−1^ K_3_[Fe (CN)_6_]. Scan rate: 0.1 V s^−1^ (**d**).

The electrocatalysis and adsorptive properties of graphene receive attention; at the same time, Au NPs could improve adsorption. Graphene and Au NPs have a synergistic effect [25]. Therefore, a composite material of graphene and Au NPs was prepared. The UV absorption of prepared Au NPs solution at a 506 nm wavelength matches previous studies (Figure S5a) [26]. The particle size distribution of the Au NPs solution, which was about 8.7 nm, is shown in Figure S5b. As illustrated in Figure 2a, Fe_3_O_4_@Au–Pt demonstrated an obvious sheet structure, and plenty of particles were uniformly distributed on the sheet structure. EDS elemental mapping pictures of GN–Au NPs are shown in Figure 2b,c; GN–Au NPs simultaneously had C and Au elements, and C elements were accompanied by Au elements. The content of C and Au elements in GN–Au NPs reached 98.97% and 1.03%, respectively (Figure S7).

The shape, current intensities, and peak potential separation of cyclic voltammetry (Figure 2d) were obtained in the presence of 0.1 mol L^−1^ KCl solution containing 1.0 mmol L^−1^ K_3_[Fe (CN)_6_]. The electrochemical signals of the GN–Au NPs modified electrodes were greatly enhanced compared with those of the electrodes modified with GN and Au NPs, respectively. Based on the above characterization, GN–Au NPs had been synthesized.

### 3.3. Development of a Graphene Nanozymes Complexes Electrochemical System

**Figure 3:**
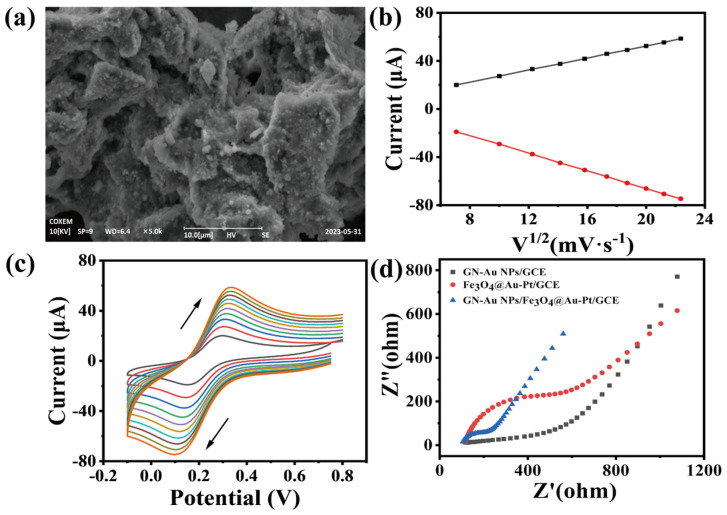
SEM images (**a**) of Fe_3_O_4_@Au–Pt/GN–Au NPs; GN–Au NPs/Fe_3_O_4_@Au–Pt/GCE modified electrodes cathode and anode linear relationship (**b**) between peak current and square root of scan rate (v^1/2^); Cyclic voltammetry curve (**c**) of GN–Au NPs/Fe_3_O_4_@Au–Pt/GCE modified electrodes in 0.1 mol L^−1^ KCl solution containing 1.0 mmol L^−1^ K_3_[Fe (CN)_6_]. Inside-to-outside scan rate: 30, 50, 100, 150, 200, 250, and 300 mV s^−1^. EIS of GN–Au NPs/GCE, Fe_3_O_4_@Au–Pt/GCE, GN–Au NPs/Fe_3_O_4_@Au–Pt/GCE in 0.1 mol L^−1^ KCl solution containing 1.0 mmol L^−1^ K_3_[Fe (CN)_6_] solution (**d**).

The SEM images of Fe_3_O_4_@Au–Pt and GN–Au NPs modified on the electrode are shown in Figure 3a. The results shown the laminated and porous structure of GN, with a large amount of spherical material on the laminated and hollow structure. EDS elemental mapping pictures of Fe_3_O_4_@Au–Pt/GN–Au NPs were shown in Figure S8a–e, demonstrating that Fe_3_O_4_@Au–Pt/GN–Au NPs simultaneously had C, Fe, O, Au, and Pt elements. The content of C, O, Fe, Pt, and Au elements in Fe_3_O_4_@Au–Pt/GN–Au NPs reached 59.21%, 39.82%, 0.40%, 0.04%, 0.53%, respectively (Figure S9). The changes in the electrical impedance occurred in various steps of electrode modifications. Electrochemical impedance spectroscopy (EIS) measurements were performed in 0.1 mol L^−1^ KCl solution containing 1.0 mmol L^−1^ K_3_[Fe (CN)_6_] solution (Figure 3b). Changes occurring in Nyquist plots indicated that electrode modification with GN–Au NPs could effectively reduce resistance. By using 0.1 mol L^−1^ KCl solution containing 1.0 mmol L^−1^ K_3_[Fe (CN)_6_], the cyclic voltammetry curve of GN–Au NPs/Fe_3_O_4_@Au–Pt/GCE modified electrodes were measured at different scan rates. Figure 3c shows the cyclic voltammetry curve of GN–Au NPs/Fe_3_O_4_@Au–Pt/GCE modified electrodes at 30, 50, 100, 150, 200, 250, and 300 mV s^−1^, respectively. The cyclic voltammetry curve plots were obtained at the scan rate, and the current values increased with the increase in the scan rate. As shown in Figure 3d, as the scan rate increased, the anode peak current and cathode peak current increased. The anode peak current (black) and the cathode peak current (red) were linearly related to the square root of the scan rate (v^1/2^), which demonstrated that the modification of GN–Au NPs/Fe_3_O_4_@Au–Pt on the electrode was the typical diffusion-controlled processes [27].

To prevent characteristic peaks from the influences of different modified electrodes, different modified electrodes were studied in PBS and H_2_O_2_ using the DPV electrochemical method. In the absence of TMB (Figure S10a), the DPV curves of Fe_3_O_4_@Au–Pt/GCE, GN–Au NPs/GCE, and GN–Au NPs/Fe_3_O_4_@Au–Pt/GCE showed no obvious characteristic peak. After adding TMB solution (Figure S10b), the DPV curves of Fe_3_O_4_@Au–Pt/GCE, GN–Au NPs/GCE, and GN–Au NPs/Fe_3_O_4_@Au–Pt/GCE showed two distinct pairs of oxidation peaks, which can be divided into +0.34 V and +0.49 V [28]. The phenomenon of the DPV curves of the different modified electrodes conforms to the mechanism of TMB oxidation. The core of the mechanism of TMB oxidation is benzidine. Benzidine possesses two easily oxidized amino groups and can be oxidized to colored products through two-electron transfer [29]. The second oxidation characteristic peak of the DPV curves was higher than the first oxidation characteristic peak. However, Figure S10a shown that the height of the oxidation characteristic peak on DPV curves of Fe_3_O_4_@Au–Pt/GCE did not conform to GN–Au NPs/GCE and GN–Au NPs/Fe_3_O_4_@Au–Pt/GCE. This result may be due to a higher electron-transfer rate of GN–Au NPs, leading to the change in height of the oxidation characteristic peak [30].

### 3.4. Optimization of the Components in the Reaction System

TMB is always used in spectrophotometric [31] and immunosorbent assays [32]. In the presence of nanozymes, TMB is oxidized by the effect of H_2_O_2_ and produces electroactive products [33]. The effectiveness of using TMB as a redox mediator was validated in Figure S11, which shown the DPV curve of different concentrations of H_2_O_2_ in the TMB comparative experiment. In the absence of a TMB electrochemical substrate, neither concentration of H_2_O_2_ had any electrochemical signal, whereas the introduction of TMB as an electrochemical substrate resulted in the appearance of an electrochemical signal. In addition, the oxidation peak current of TMB decreased with increasing H_2_O_2_ concentration. The decrease in oxidation current was caused by the depletion of TMB. Therefore, this result suggests that TMB can be used as an effective redox mediator for improving the sensitivity and lowering the detection limit. Therefore, TMB is known as a detection medium in various electrochemical biosensors [34]. Taking advantage of this property, electrochemical sensors using H_2_O_2_ and TMB as reaction substrates were constructed. The effects of the pH value and TMB concentration on electrochemical signals were studied to make Fe_3_O_4_@Au–Pt display the highest activity. Figure S12a shown that Fe_3_O_4_@Au–Pt displayed the best activity at pH 7.4. Because the TMB has electrical activity, electrochemical signals tend to increase as the TMB concentration increases. As illustrated in Figure S12b, when the TMB concentration was larger than 1.0 mmol L^−1^, the growth rate of the electrochemical signal slowed down gradually. As shown in Figure S12c, when the reaction time exceeded 5 min, the electrochemical signal gradually decreased. Therefore, Fe_3_O_4_@Au–Pt was capable of the best activity at a pH value of 7.4, a TMB concentration of 1.0 mmol L^−1^, and a reaction time of 5 min.

### 3.5. Detection of H_2_O_2_ by Graphene Complexes Electrochemical System

**Figure 4:**
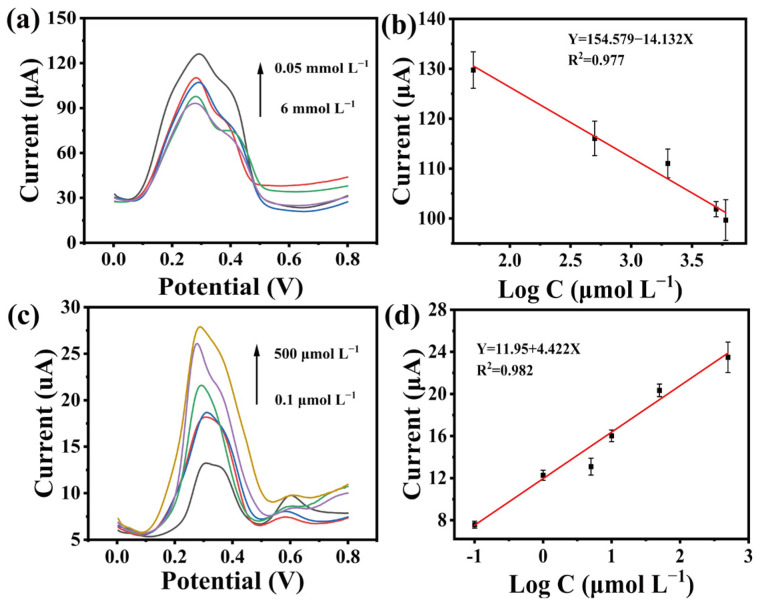
The electrochemical detection results of H_2_O_2_ and ETH using the MRMEC method. (**a**) DPV curves of TMB corresponding to H_2_O_2_ with different dosages. (**b**) The linear relationship between DPV current and the logarithm concentration of H_2_O_2_ from 0.05 mmol L^−1^ to 6 mmol L^−1^. Error bars represent the standard error of the mean (n = 3). (**c**) DPV curves of TMB correspond to ETH with different dosages. (**d**) The linear relationship between DPV current and the logarithm concentration of ETH from 0.1 μmol L^−1^ to 500 μmol L^−1^. Error bars represent the standard error of the mean (n = 3).

It can be seen from Figure 4a that the oxidation peak current of TMB decreased with increasing H_2_O_2_ concentration. The decrease in the TMB oxidation current peak was due to the consumption of TMB by H_2_O_2_, resulting in a decrease in the number of TMB molecules that can be electrochemically oxidized at the electrode. Different concentrations of H_2_O_2_ solutions were detected using the optimized parameters. In Figure 4b, a linear relationship was obtained by converting the H_2_O_2_ concentration to a logarithmic value. The linear relationship indicated that under the synergy of both Fe_3_O_4_@Au–Pt and GN–Au NPs, H_2_O_2_ as substrate reaction, TMB accelerates the oxidation process. Each data point represents the average of three parallel experiments. The linear response range for the current using modified electrochemical sensors was 0.05 to 6.0 mmol L^−1^, and the working equation was Y = 154.579 − 14.132X (R^2^ = 0.977, n = 3), Y and X were the current peak height using DPV and the logarithmic of concentration of H_2_O_2_, respectively. The standard deviation of the blank sample was then used to calculate the limit of detection (LOD), and the LOD was determined to be 0.003 mmol L^−1^.

### 3.6. Development of MRMEC for ETH

According to the above information about Fe_3_O_4_@Au–Pt and GN–Au NPs electrochemical sensors, optimal reaction conditions for Fe_3_O_4_@Au–Pt and GN–Au NPs electrochemical sensors were determined. Therefore, H_2_O_2_ is used as an intermediate, and TMB is used as the signal substance to detect ETH [35]. It must be accepted that ETH is an organophosphorus pesticide and acts as an acetylcholinesterase (AChE) inhibitor. Based on the enzyme inhibition principle, a biosensor was developed to detect ETH. In the absence of ETH, AChE, acetylcholine (ACh), and choline oxidase (CHOx) in the suitable reaction environment could react smoothly and produce H_2_O_2_. Consequently, in the opposite situation, the reaction capability of ACh, AChE, and CHOx affected by ETH leads to a change in the content of H_2_O_2_. The change in the content of H_2_O_2_ could influence the oxidation peak current of TMB. The above-mentioned can lead to the conclusion that the oxidation peak current of TMB increases with increasing ETH concentration. In light of the variation in the oxidation peak current of TMB, MRMEC for ETH will be readily developed.

Different enzymes are very sensitive to their environment, and the reaction environment has been determined by the related literature. Related literature shows that the optimal concentrations for ACh, AChE, and CHOx are 5.0 mmol L^−1^, 500 U L^−1^, and 2000 U L^−1^, respectively [36]. 

As shown in Figure 4c, with the continuous addition of ETH, the DPV current of TMB gradually increased. This explained that the inhibition of AChE activity by ETH led to a decrease in the H_2_O_2_ hydrolysis rate and TMB oxidation rate. The relationship between DPV current and concentration of ETH is shown in Figure 4d. The oxidation peak current of TMB and the logarithm concentration of ETH exhibited a good linear relationship (R^2^ = 0.982) from 0.1 to 500 μmol L^−1^, and the working equation was Y = 4.422X + 11.950 (n = 3). The LOD was calculated to be 2.01 nmol L^−1^, which met the maximum ETH residue limits for pesticides in food as stipulated in the Chinese national standard [21]. By fitting two sets of farthest data points within the error bar, the error of the LOD was estimated to be 5.82%, revealing a good reproducibility and stability with RSD (relative standard deviation) of 2.92–6.17% (Table S1).

Compared to the bare electrode (LOD, 0.32 μmol L^−1^), the MRMEC method showed a lower LOD value with enhanced signal intensity (Figure S13), which can be attributed to the good catalytic property of Fe_3_O_4_@Au–Pt and a large reaction area of GN–Au NPs. Moreover, the electrochemical sensor is not easily disturbed by factors such as color development in the food matrices. As shown in Table 1, the method has a comparable detection sensitivity and wide linear range compared to other methods reported in the literature. 

### 3.7. MRMEC for ETH Detection Performance Studies

**Figure 5:**
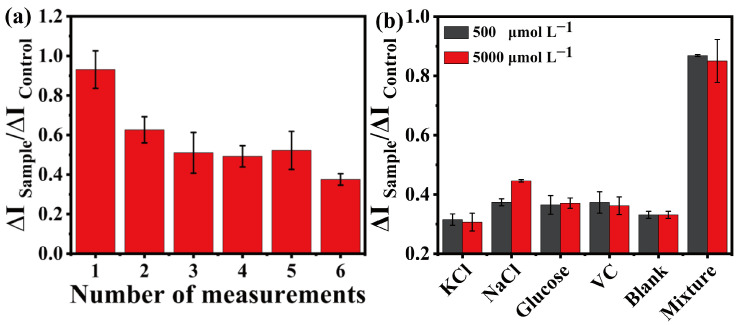
Reusability tests performed (**a**). Signal ratio of sample and control of Fe_3_O4@Au–Pt and GN–Au NPs electrochemical sensors detection for 500 μmol L^−1^ (red) and 5000 μmol L^−1^ (dark gray) interfering substances, mixture (a mixture of 50 μmol L^−1^ ETH and 500 or 5000 μmol L^−1^ four interfering substances), and blank, respectively (**b**).

The repeated cleaning for MRMEC may damage the modifiers of electrochemical sensors. Therefore, the reusability of the modified electrode was explored. Under the optimized working conditions, the MRMEC preserved 52 ± 10% of the original response signal after five tests (Figure 5a). As the electrode was reused with undesirable results, the samples were detected with the newly-decorated electrodes. In the detection, the performance of the electrochemical sensor will be suppressed by the interference of some accompanying substances in the samples. Therefore, the cross-selectivity of the sensor was further studied. These interfering substances were KCl, NaCl, glucose, VC (vitamin C), mixture (a mixture of 50 μmol L^−1^ ETH and four interfering substances), and blank. The concentration of interfering substances was 10 or 100 times that of the target detector. The mixture sample contained 50 μmol L^−1^ ETH and four interfering substances (500 or 5000 μmol L^−1^). The mixture showed a higher ratio signal (ΔI_sample_/ΔI_control_) than the blank group and the experimental group containing only a single interfering substance (Figure 5b). In this regard, MRMEC exhibited acceptable detection selectivity.

Except for ETH, other pesticides inhibit AChE [42]. Therefore, electrochemical sensors must assess the degree of response of the detected signal for other pesticides. Therefore, 50 μmol L^−1^ ETH, ACE (acetamiprid), IMI (imidacloprid), and CBZ (carbendazim) were selected for evaluation. CBZ, a systemic, broad-spectrum benzimidazole fungicide, is widely used to control fungal diseases in agricultural products [43]. Thus, CBZ is unable to inhibit AChE, and the signal ratio of the CBZ sample and control was low. In contrast, other pesticides, such as ETH, ACE, and IMI, are able to inhibit AChE activity and have a high signaling ratio (Figure S14).

### 3.8. Sample Measurement

The MRMEC method established in this study was used in the spiked recovery test of mangoes, and the results are shown in Table 2. Five different concentrations (0.05, 0.5, 5, 50, and 500 μmol L^−1^) of ETH in the middle range of the linear range were sprayed on the surface of mangoes. The sample recovery ratio varied from 92.18% to 110.72%. The coefficient of variations (CV) values were 2.88% to 15.97%, indicating that this MRMEC had good accuracy and reliability in detecting ETH. 

## 4. Conclusions

In summary, the MRMEC was proposed for ETH detection with Fe_3_O_4_@Au–Pt and GN–Au NPs modified GCE. Fe_3_O_4_@Au–Pt exhibited excellent peroxidase-like activity, large specific surface area, and high enzymatic activity. GN–Au NPs have good electrochemical signal enhancement effects. An electrochemical biosensor with color-interference resistance and rapid response was constructed using the multienzyme reaction system. The sensor achieved ETH specificity and high sensitivity detection with the LOD of 2.01 nmol L^−1^. This work not only extends the application of nanozymes from colorimetric analysis to electrochemistry but also expands the establishment of the method for ETH to other pesticides. It provides appropriate guidance for the design of nanozyme electrochemical sensors and the detection of other pesticides, especially for the determination of the total concentration of organophosphate pesticides in real samples.

## Figures and Tables

**Table 1:** MRMEC with other reported methods for the detection of ETH.

Methods	Linear Ranges (mol L^−1^)	LOD (mol L^−1^)	LOQ (mol L^−1^)	References
SERS with HKUST-1(Cu) ^1^	6.92 × 10^−9^–6.92 × 10^−5^	9.62 × 10^−10^	2.92 × 10^−9^	[37]
Ion-pairing LC-MS/MS ^2^	3.30 × 10^−8^–3.30 × 10^−6^	3.30 × 10^−8^	8.25 × 10^−8^	[38]
Negative-ion LC-MS/MS ^3^	6.92 × 10^−8^–6.92 × 10^−7^	2.07 × 10^−7^	6.21 × 10^−7^	[39]
Hydrophilicity GC-MS/MS ^4^	3.50×10^−7^–1.3×10^−6^	1.70 × 10^−7^	3.40 × 10^−7^	[40]
EC with Polyaniline@SnO_2_ ^5^	6.90 × 10^−11^–3.4 × 10^−8^	3.30 × 10^−11^	1.00 × 10^−10^	[41]
MRMEC	1.00 × 10^−7^–5.00 × 10^−4^	2.01 × 10^−9^	6.00 × 10^−9^	This work

^1^ SERS with HKUST-1(Cu): flexible SERS strip based on HKUST-1(cu)/biomimetic antibodies composite multilayer. ^2^ Ion-pairing LC-MS/MS: an ion-pairing liquid chromatography/tandem mass spectrometric method. ^3^ Negative-ion LC-MS/MS: negative-ion mode liquid chromatography with tandem mass spectrometry. ^4^ Hydrophilicity GC-MS/MS: hydrophilic interaction chromatography tandem mass spectrometry. ^5^ EC with Polyaniline@SnO_2_: electrochemical sensor based on a polyaniline-modified SnO_2_.

**Table 2:** Recoveries of ETH from mangoes at different concentration levels (n = 3).

Sample	Added (μmol L^−1^)	Found (μmol L^−1^)	Recovery (%)	CV (%)
Mangoes	0.05	0.052 ± 0.005	104.13	9.79
	0.50	0.55 ± 0.02	110.72	2.88
	5.00	5.5 ± 0.8	110.59	15.97
	50.00	50 ± 2	99.64	3.24
	500.00	460 ± 20	92.18	4.81

## Data Availability

Data are contained within the article and supplementary materials.

## Supplementary Materials

The following supporting information can be downloaded at https://www.mdpi.com/article/10.3390/bios14020062/s1: Figure S1: Schematic diagram of the fabrication of Fe_3_O_4_@Au–Pt; Figure S2: EDS spectrogram of Fe_3_O_4_@Au–Pt; Figure S3: UV−vis curves of TMB, TMB+ Fe_3_O_4_@Au–Pt, TMB + H_2_O_2_, TMB + H_2_O_2_ + Fe_3_O_4_@Au–Pt (a). The different modified electrodes in PBS (0.1 mol L^−1^, pH = 7.4) (b) and H_2_O_2_ (6 mmol L^−1^) (c) influence the DPV curves. Current–time curve of Fe_3_O_4_@Au–Pt/CGE in H_2_O_2_ (d); Figure S4: (a) Calibration curve for H_2_O_2_ detection; (b) The linear relationship between absorbance at 652 nm and the logarithm concentration of H_2_O_2_ from 0.05 mmol L^−1^ to 6 mmol L^−1^. Error bars represent the standard error of the mean (n = 3); Figure S5: Particle size distribution of Au NPs (a); Absorption spectrum of Au NPs (b); Figure S6: Schematic diagram of the fabrication of GN–Au NPs; Figure S7: EDS spectrogram of Fe_3_O_4_@Au–Pt; Figure S8: EDS Fe (a), Au (b), Pt (c), O (d), and C (e) elemental mapping pictures of Fe_3_O_4_@Au–Pt/GN-Au NPs; Figure S9: EDS spectrogram of Fe_3_O_4_@Au–Pt/GN–Au NPs; Figure S10: The different modified electrodes in PBS (0.1 mol L^−1^, pH = 7.4) (a) and H_2_O_2_ (6 mmol L^−1^) and TMB (1 mmol L^−1^) (b) influence the DPV curves; Figure S11: DPV curves of GN–Au NPs/Fe_3_O_4_@Au–Pt/GCE in 0.05 mmol L^−1^ (a) and 6 mmol L^−1^ (b) concentrations of H_2_O_2_. Figure S12: The pH (a) on the response currents in 1 mmol L^−1^ TMB solution, TMB concentration (b) on the response currents with PBS (0.1 mol L^−1^, pH = 7.4), and reaction time (c) on the response currents influence with 1 mmol L^−1^ TMB solution and PBS (0.1 mol L^−1^, pH = 7.4); Figure S13: (a) DPV curves of TMB correspond to ETH with different dosages (from 0.1 μmol L^−1^ to 500 μmol L^−1^) using bare electrode. (b) The linear relationship between DPV current and the logarithm concentration of ETH corresponding to (a). Error bars represent the standard error of the mean (n = 3); Figure S14: Signal ratio of sample and control of Fe3O4@Au–Pt and GN–Au NPs electrochemical sensors detection for 50 μmol L^−1^ ETH (Ethephon), ACE (Acetamiprid), IMI (Imidacloprid), CBZ (Carbendazim), and Blank; Table S1: RSD value of each linear concentration gradient (n = 3).
