# Supplementary material for: A Multienzyme Reaction-Mediated Electrochemical Biosensor for Sensitive Detection of Organophosphorus Pesticides
Source: Biosensors (Basel). 2024 Jan 24;14(2):62. doi: 10.3390/bios14020062 (PMC10886554; doi:10.3390/bios14020062)
Supplement: Supplementary file 1 [file biosensors-14-00062-s001.zip › biosensors-2755342-supplementary.pdf]

Supplementary Materials

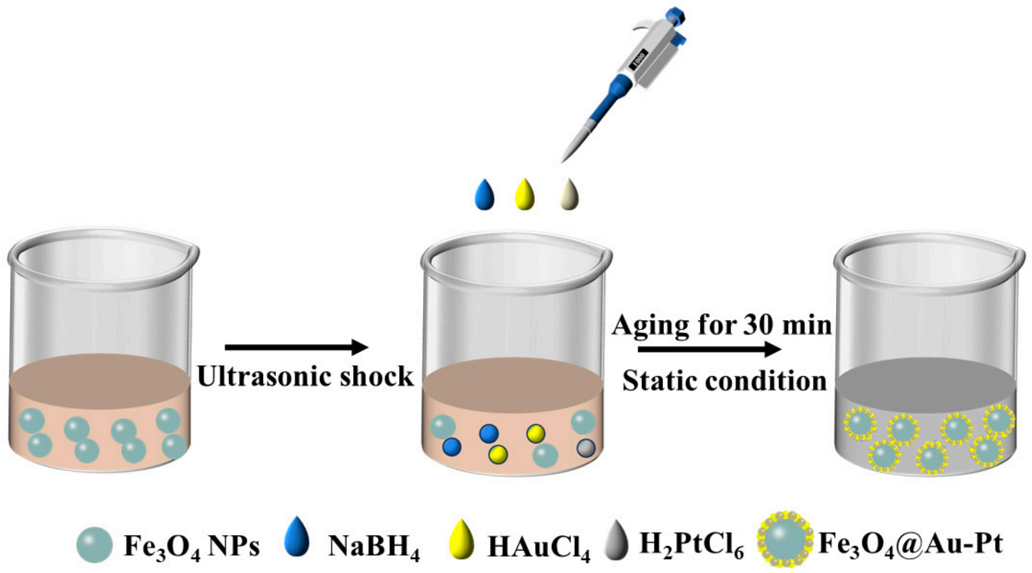

Figure S1. Schematic diagram of the fabrication of Fe_3O_4@Au-Pt.

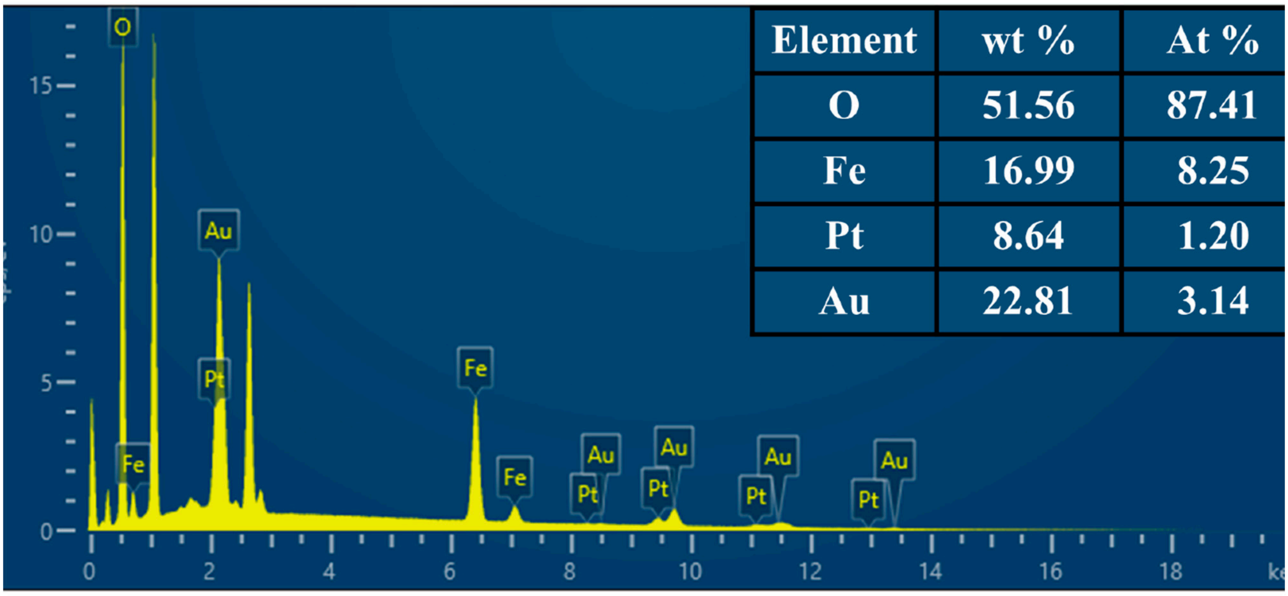

Figure S2. EDS spectrogram of Fe_3O_4@Au-Pt.

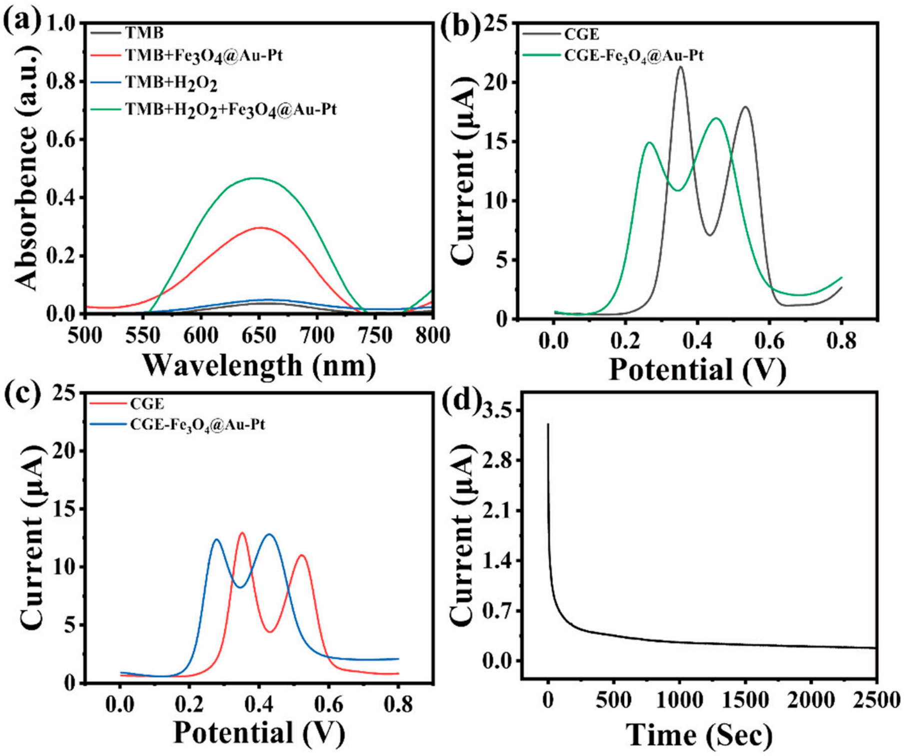

Figure S3. UV−vis curves of TMB, TMB+ Fe_3O_4@Au-Pt, TMB+H_2O_2, TMB+H_2O_2+ Fe_3O_4@Au-Pt **(a)**. The different modified electrodes in PBS (0.1 mol L^{-1}, pH=7.4) **(b)** and H_2O_2 (6 mmol L^{-1}) **(c)** influence the DPV curves. Current-time curve of Fe_3O_4@Au-Pt/CGE in H_2O_2**(d)**.

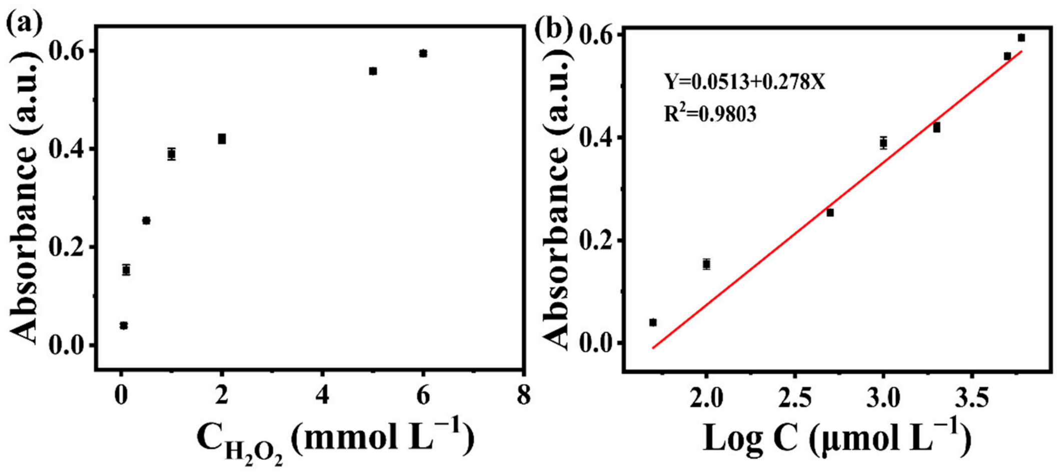

Figure S4. **(a)** Calibration curve for H_2O_2 detection; **(b)** The linear relationship between absorbance at 652 nm and the logarithm concentration of H_2O_2 from 0.05 mmol L^{-1} to 6 mmol L^{-1}. Error bars represent the standard error of the mean (n=3).

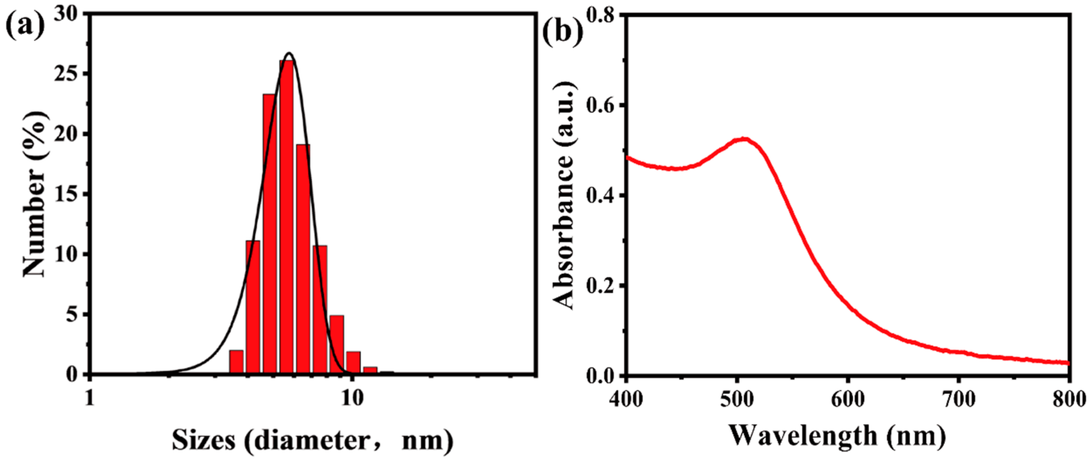

Figure S5. Particle size distribution of Au NPs **(a)**; Absorption spectrum of Au NPs **(b)**.

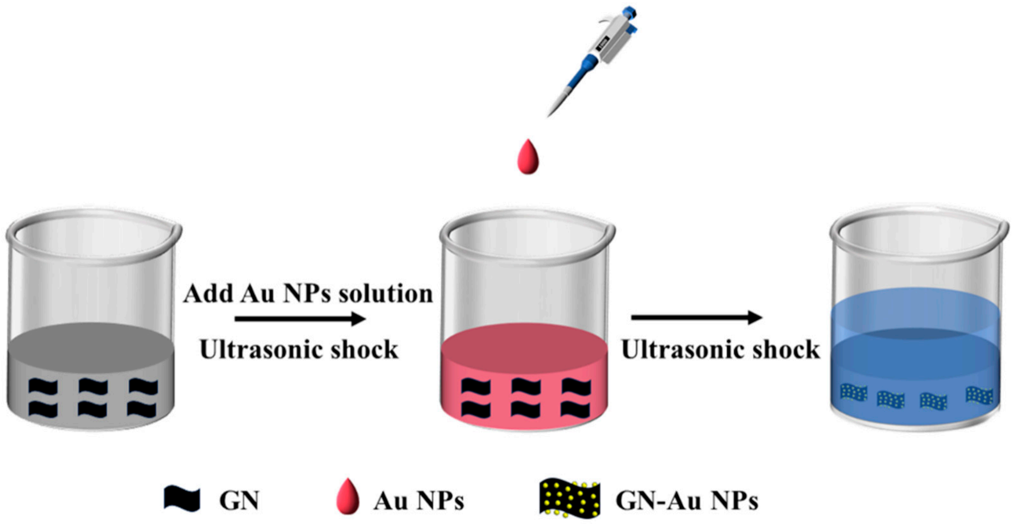

Figure S6. Schematic diagram of the fabrication of GN-Au NPs.

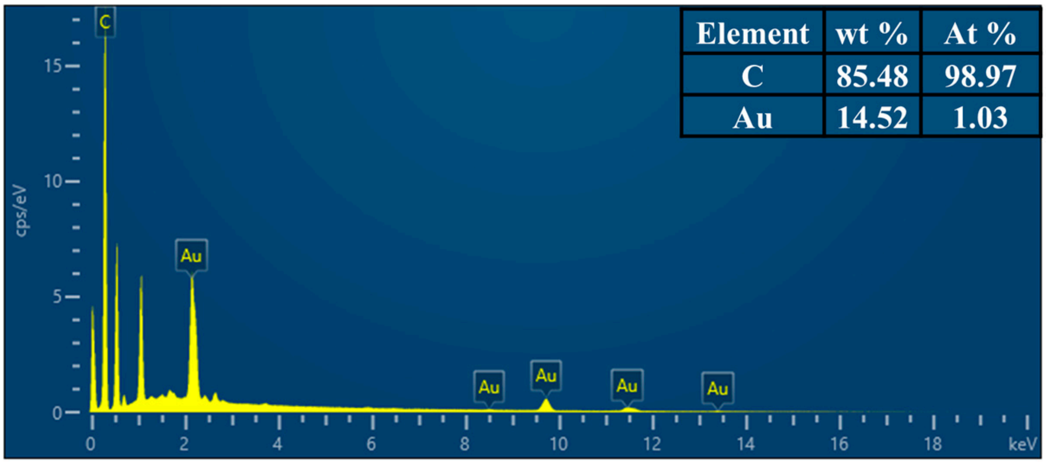

Figure S7. EDS spectrogram of Fe_3O_4@Au-Pt.

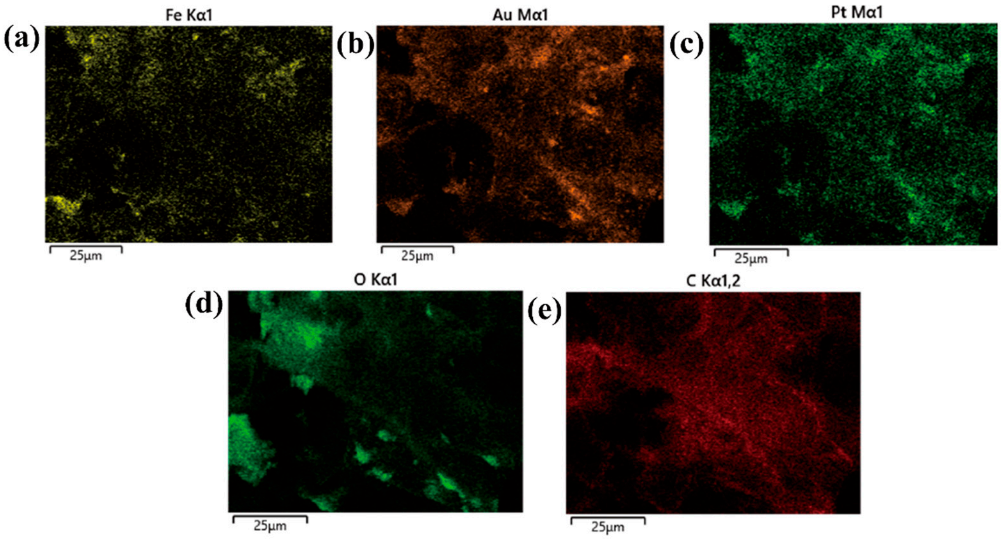

Figure S8. EDS Fe (a), Au (b), Pt (c), O (d), and C (e) elemental mapping pictures of Fe_3O_4@Au-Pt/GN-Au NPs.

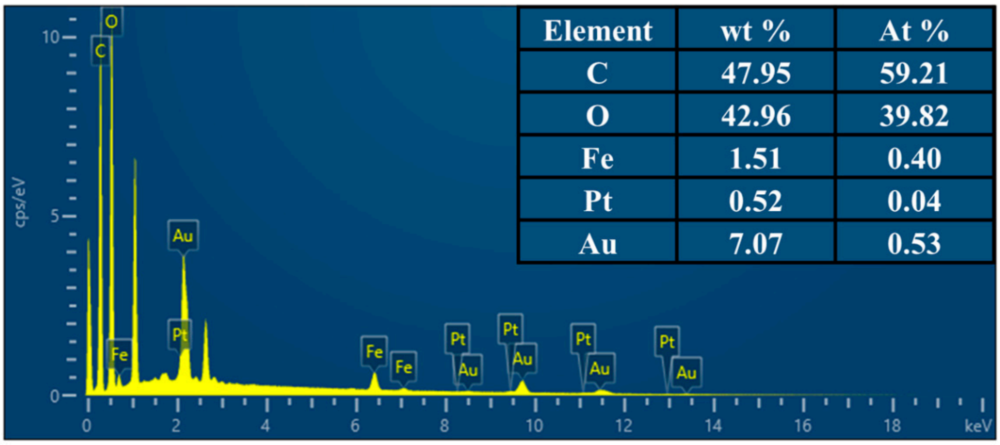

Element	wt %	At %
C	47.95	59.21
O	42.96	39.82
Fe	1.51	0.40
Pt	0.52	0.04
Au	7.07	0.53

Figure S9. EDS spectrogram of Fe_3O_4@Au-Pt/GN-Au NPs.

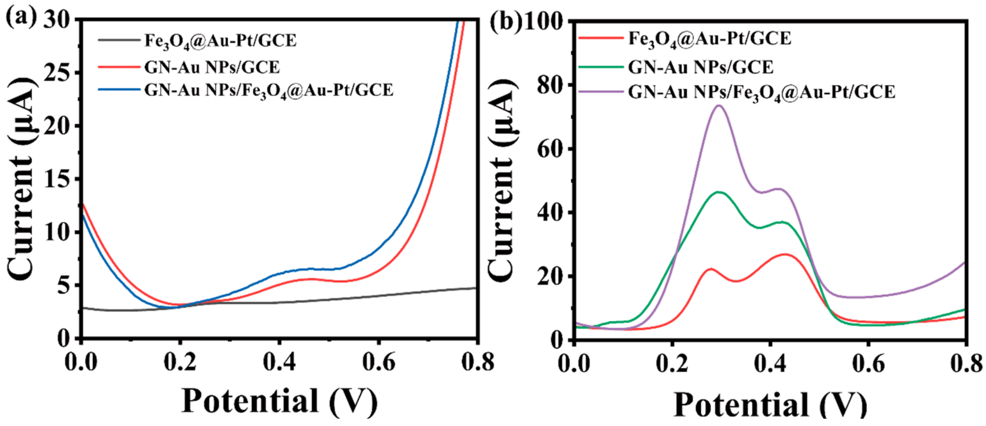

Figure S10. The different modified electrodes in PBS (0.1 mol L^{-1}, pH=7.4) **(a)** and H_2O_2 (6 mmol L^{-1}) and TMB (1 mmol L^{-1}) **(b)** influence the DPV curves.

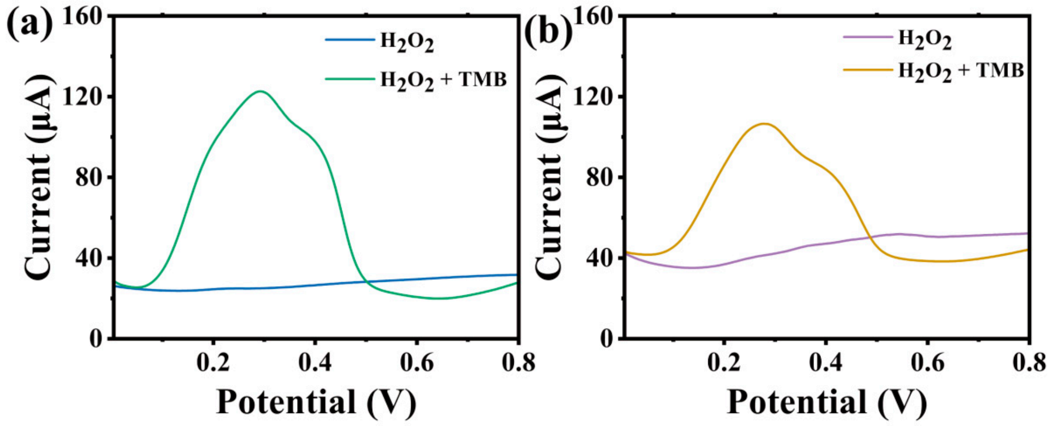

Figure S11. DPV curves of GN-Au NPs/Fe_3O_4@Au-Pt/GCE in 0.05 mmol L^{-1} **(a)** and 6 mmol L^{-1} **(b)** concentrations of H_2O_2.

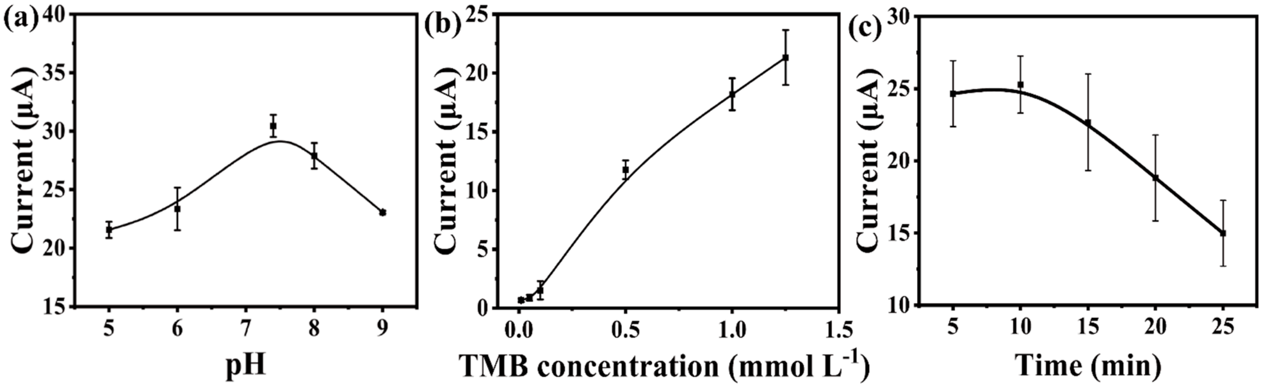

Figure S12. The pH **(a)** on the response currents in 1 mmol L^{-1} TMB solution, TMB concentration **(b)** on the response currents with PBS (0.1 mol L^{-1}, pH=7.4), and reaction time **(c)** on the response currents influence with 1 mmol L^{-1} TMB solution and PBS (0.1 mol L^{-1}, pH=7.4). Error bars represent the standard error of the mean (n=3).

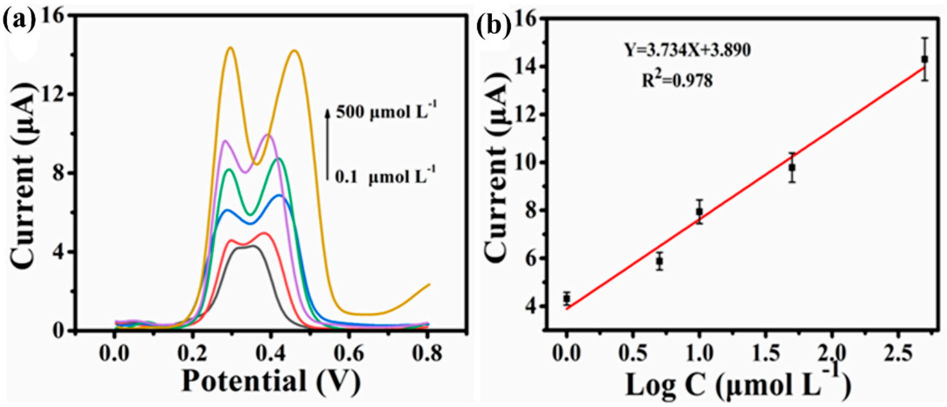

Figure S13. **(a)** DPV curves of TMB correspond to ETH with different dosages (from 0.1 μmol L^{-1} to 500 μmol L^{-1}) using bare electrode. **(b)** The linear relationship between DPV current and the logarithm concentration of ETH corresponding to (a). Error bars represent the standard error of the mean (n=3).

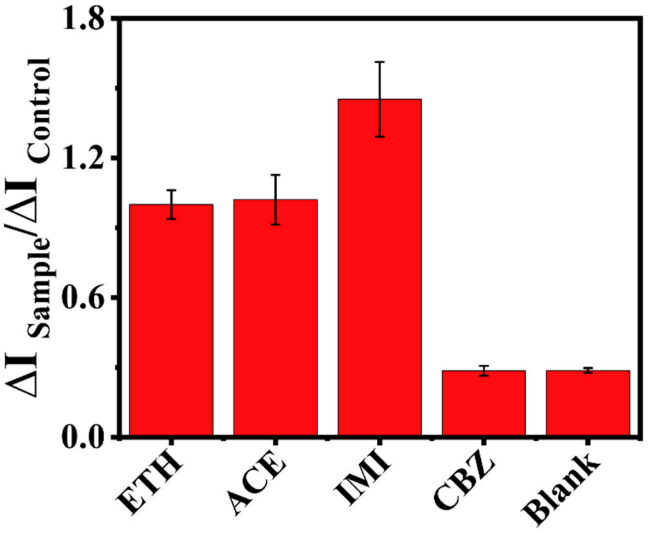

Figure S14. Signal ratio of sample and control of Fe_3O_4@Au-Pt and GN-Au NPs electrochemical sensors detection for 50 μmol L^{-1} ETH (Ethephon), ACE (Acetamiprid), IMI (Imidacloprid), CBZ (Carbendazim), and Blank. Error bars represent the standard error of the mean (n=3). Error bars represent the standard error of the mean (n=3).

Table S1. RSD value of each linear concentration gradient (n=3).

**Linear concentration (μmol L^{-1})**	0.1	1	5	10	50	500
**RSD (%)**	4.36	3.86	6.12	3.44	2.92	6.17
